# Endovascular thrombectomy for subterminal internal carotid artery occlusions: clinical and radiological heterogeneity in a nationwide registry

**DOI:** 10.1007/s00234-026-04109-2

**Published:** 2026-07-14

**Authors:** Emma Hall, Björn M. Hansen, Alex Szolics, Teresa Ullberg, Johan Wassélius

**Affiliations:** 1 Department of Clinical Science, Lund University, Lund, Sweden; 2 Department of Neuroradiology, Skåne University Hospital, Lund, Sweden; 3 Department of Clinical Neuroscience, Karolinska Institutet, Stockholm, Sweden; 4 Department of Neurology, Skåne University Hospital, Malmö, Sweden

**Keywords:** Endovascular thrombectomy, subterminus intracranial internal carotid artery occlusion, computed tomography angiography, diagnostic accuracy, neuroradiology, imaging characteristics, outcomes, treatment delays

## Abstract

**Purpose:**

Subterminal intracranial Internal Carotid Artery (ICA-I) occlusion is a lesser-known stroke subtype which may delay access to endovascular thrombectomy (EVT). We compared clinical presentation, imaging characteristics, treatment delays, outcomes, and diagnostic accuracy between EVT-treated patients with ICA-I and carotid terminus (ICA-T) occlusions.

**Methods:**

All EVT-treated patients with intracranial ICA occlusions registered in two Swedish national quality registers (2016–2022) were included. Outcomes included pre- and postoperative National Institutes of Health Stroke Scale (NIHSS), successful recanalization, and 90-day modified Rankin Scale. Perfusion images and original radiology reports from one comprehensive stroke center were analyzed to assess the relationship between collateral anatomical variants, stroke severity, perfusion deficits, and diagnostic accuracy.

**Results:**

Among 6163 EVT-treated patients, 356 had ICA-I occlusions and 657 had ICA-T occlusions. Median baseline NIHSS was lower in ICA-I occlusions (17 vs. 19), but variability in NIHSS and modified Alberta Stroke Program Early CT Score were higher (both *p* < 0.001). ICA-I occlusions were associated with EVT treatment delays, but recanalization rates, 24-hour NIHSS, and 90-day functional outcomes were similar between groups. In exploratory hypothesis-generating sub-group analyses of ICA-I occlusions, anterior collateral variants were associated with stroke severity, and fetal-type posterior cerebral artery variants were associated with larger perfusion deficits. In a sub-group analysis, ICA-I occlusions were correctly identified in 31% of baseline radiology reports compared with 57% of ICA-T occlusions.

**Conclusion:**

ICA-I occlusions show greater clinical and radiological heterogeneity, are frequently underrecognized on baseline imaging, and are associated with longer treatment delays than ICA-T occlusions. Increased awareness may improve acute stroke triage and reduce delays to treatment.

## Introduction

Acute ischemic stroke caused by anterior circulation large vessel occlusion remains a major cause of death and long-term disability worldwide [1]. Endovascular thrombectomy (EVT) is the standard of care for anterior circulation large vessel occlusions and in the five landmark randomized controlled trials of 2015 approximately 20% of the included patients were treated for an internal carotid artery (ICA) occlusion [2]. ICA occlusions are, because of their large vascular territory, associated with particularly severe clinical presentations, large infarct cores, and severe hemorrhagic complications [3], despite rapid reperfusion therapy.

**Fig. 1:**
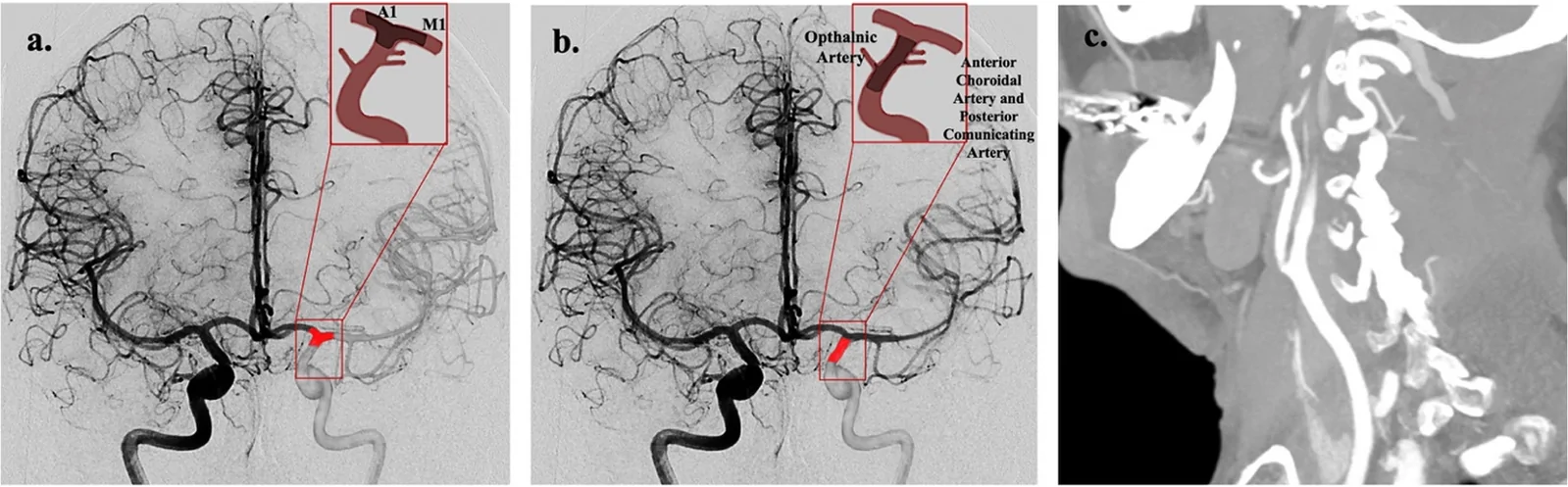
(**a**) Illustration of an ICA-T occlusion with thrombus in the distal ICA, M1 and A1 segments; (**b**) illustration of an ICA-I occlusion with thrombus in the distal ICA with involvement of the posterior communicating artery, the ophthalmic artery, and the anterior choroidal artery but preserved blood flow in the middle cerebral artery via the anterior axis; and (**c**) CTA with sagittal view of the gradually diminishing contrast density observed in the proximal ICA in I and T occlusions

Intracranial ICA occlusions are commonly subdivided into ICA-I, limited to the intracranial ICA below the carotid terminus, or ICA-T, involving the carotid terminus with occlusion of both the anterior cerebral artery (ACA) and middle cerebral artery (MCA) origins (Fig. 1a-b) [4–6]. Although often grouped together, these subtypes differ in collateral flow capacity and may therefore present with distinct clinical, radiological, and functional profiles. Previous studies have reported conflicting results regarding stroke severity and recanalization rates [4–6] and direct comparisons between the two occlusion types remain limited.

Accurate diagnosis of ICA occlusions can be challenging. Contrast stagnation proximal to the occlusion may produce a pseudo-occlusion appearance (Fig. 1c), potentially leading to misclassification as proximal ICA stenosis, occlusion or dissection [7, 8]. ICA-I occlusions may be overlooked on CTA, particularly in the presence of preserved collateral circulation and the pseudo-occlusion phenomenon [7, 8]. Although an ICA-I occlusion may at times appear less dramatic on imaging or exam, it can endanger as much brain tissue as an ICA-T occlusion, depending on collateral efficacy. Accurate and rapid identification of ICA-I occlusions is thus important for improving patient outcome.

This study aimed to characterize the clinical presentation, imaging findings, and outcomes of EVT-treated ICA-I occlusions compared with ICA-T occlusions. Secondary, the study also aimed to explore the influence of collateral anatomical variants on stroke severity and perfusion deficits, and to assess the accuracy of baseline CTA interpretation.

## Methods

## Study design and data sources

Data were obtained from the two Swedish quality registries for stroke care: the Riksstroke Register (XXX) [9] and the Swedish Endovascular Thrombectomy for Acute Ischemic Stroke Register (EVAS) [10]. We included all patients treated with EVT for intracranial ICA occlusions in Sweden between 2016 and 2022 who were registered in both databases. Radiological and procedural variables were collected from EVAS, which compiles detailed procedural data from all seven comprehensive stroke centers performing EVT in Sweden. Complementary clinical data, including pre- and post-stroke functional status and outcomes, were obtained from Riksstroke, which covers all 72 hospitals providing acute stroke care nationwide.

Baseline perfusion and angiographic imaging data were only available for patients treated at Lund University Hospital 2016–2024. Accordingly, the sub-analysis was performed including all patients with ICA-I occlusions treated with EVT at this center, assessing differences in pre-EVT NIHSS and perfusion deficits in relation to predefined anatomical variants of the anterior and posterior circulation.

### Clinical and radiological outcomes

The studied outcomes were median NIHSS and its variance before and after EVT (with higher variance indicating that the NIHSS values are further spread out from the mean), successful recanalization, early neurological deterioration, and functional independence after 90 days.

Intracranial ICA occlusions were subdivided into ICA-I and ICA-T and were defined by their distal and proximal ends on digital subtraction angiography (DSA) prior to EVT, in line with earlier studies [4]. Patients with intracranial ICA occlusions involving the ICA terminus, thereby blocking the proximal ACA and MCA were defined as T-occlusions. Patients with intracranial ICA occlusions involving the posterior communicating artery (PComA), anterior choroidal, or ophthalmic arteries, but sparing the MCA/ACA origins, were classified as ICA-I occlusions. The vessel occlusion classification was reviewed by two interventional neuroradiologists for all patients in the image analysis subset of patients treated at Lund University Hospital 2016–2022. Patients with multiple occlusions were not included in this part of the study.

The modified Alberta Stroke Program Early CT Score (ASPECTS) is a 10-point scoring system that quantifies the extent of early ischemic damage within specific regions of the MCA territory [11]. In our study, the extent of early infarction was categorized into mASPECTS groups based on the infarct classification available in EVAS: mASPECTS 10: no evidence of ischemic injury; mASPECTS 7–9: infarct involving less than one-third of the MCA territory or infarction in the basal ganglia; mASPECTS 5–6: infarct involving less than one-third of the MCA territory with infarction also present in the basal ganglia; and mASPECTS 0–4: infarct affecting more than one-third of the MCA territory.

Successful recanalization was defined as a modified Thrombolysis in Cerebral Infarction (mTICI) score of 2b–3 at the end of the EVT procedure, and early neurological deterioration was defined as an increase in NIHSS of a minimum of 4 points and/or death within 24 h. Functional outcome was assessed using the modified Rankin Scale (mRS) where mRS 0–2 at 90 days was defined as functional independence.

### Anatomical variants, NIHSS, and perfusion imaging

Anatomical variants of the circle of Willis in patients with ICA-I occlusions were defined based on computed tomography angiography (CTA). Variants in the anterior portion of the Circle of Willis were divided into (1) unbroken/patent anterior axis or (2) broken anterior axis, i.e. hypoplastic or absent A1 segment or anterior communicating artery ipsi- or contralateral to the occlusion. Variants in the posterior circulation were divided into (3) unbroken anterior axis with ipsilateral fetal-type posterior cerebral artery (PCA) and (4) unbroken anterior axis with ipsilateral non-fetal type PCA, in line with classifications in earlier studies [12]. Measurements included NIHSS before EVT, and CT perfusion data including core defined as relative cerebral blood flow under 30% (CBF < 30) and time to maximal enhancement > 6 s (Tmax>6s) volumes which were assessed using RAPID (iSchemaView, Menlo Park, CA).

### Validation of initial radiology reports

As a secondary outcome, we assessed the accuracy of baseline CTA reports by the primary on-call radiologist among patients with ICA-I occlusions. The reports were only available from the local health care region (Skåne) which consists of 9 acute care hospitals. For each ICA-I case, the next consecutive EVT-treated ICA-T case was selected by manual review of PACS data, as a control. Patients with tandem occlusions, multiple occlusions, or dissections were excluded.

Experience level for the author of the original radiology report was noted as either neuroradiologist, general radiologist or radiology resident. The original CTA baseline radiology report interpretations were compared with a consensus assessment based on the CTA and DSA findings of the treating interventional neuroradiologist. This comparison was performed jointly by a neurointerventional fellow and a senior neurointerventionist with 25 years of experience. Reports were classified as: (1) correct intra- and extracranial assessment (2) incorrect intracranial assessment, (3) incorrect extracranial assessment, and (4) incorrect intra- and extracranial assessment.

### Statistical analysis

Statistical analyses were conducted using IBM SPSS Statistics version 30. Baseline characteristics were presented as medians with interquartile ranges (IQR), or as simple proportions. A two-sided p-value < 0.05 was considered statistically significant.

Mann-Whitney U-tests were used to compare median NIHSS values, age, and time from stroke onset to groin puncture. Because one of the study hypotheses was that ICA-I occlusions present with greater clinical heterogeneity than ICA-T occlusions, variability in NIHSS scores was analyzed using variance and Levene’s test. The association between occlusion location and functional independence (mRS 0–2) was analyzed using univariable logistic regression and multivariable logistic regression adjusted for age, sex, mASPECT, hypertension, administration of intravenous thrombolysis (IVT), and NIHSS before EVT.

Missing mRS follow-up data were addressed using multiple imputation by chained equations with the following predicting variables: mRS before stroke onset, age, sex, diabetes, NIHSS score before and 24 h after EVT, mTICI score at the end of the procedure, and mASPECTS. Twenty complete datasets were generated and the estimates from each imputation were combined using Rubin’s rule.

The difference in pre-EVT NIHSS between ICA-I occlusions with different anatomical variants were presented as median with IQR and analyzed with Mann-Whitney U-test. CT perfusion data were presented as means.

The results in the validation of initial radiology reports were presented as simple proportions.

## Results

### Clinical and radiological outcomes

Out of 6163 patients with acute ischemic stroke registered in both Riksstroke and EVAS during 2016 and 2022, 3730 were treated with EVT due to an anterior circulation large vessel occlusion. Of these, 356 patients (10%) presented with an ICA-I occlusion and 657 (18%) with an ICA-T occlusion and therefore included in the study. The review of the vessel occlusion classification for patients in the image analysis subset of patients treated at Lund University Hospital 2016–2022 showed that all cases were correctly classified.

Baseline data on patient and stroke characteristics are shown in Table 1. The median age was 75 years for both groups. Female sex was less frequent in the ICA-I group (44%) compared to the ICA-T group (51%), and hypertension was more common in patients with ICA-I occlusions (69%) compared to ICA-T occlusions (60%).

Baseline mASPECTS scores differed significantly across the two occlusion groups (*p* < 0.019). Patients in the ICA-I group had a higher proportion of mASPECTS 10 (47%, ICA-T: 39%). At the same time, the ICA-I group also showed a slightly higher proportion of severe infarcts (mASPECTS 0–4: 6%), compared with patients with ICA-T occlusions (4%).

A significant difference was also observed in infarct location. ICA-I occlusions resulted in a higher frequency of posterior circulation infarcts at baseline (9%) as compared to ICA-T occlusions (5%, *p* = 0.037).

There were significant differences in median time from stroke onset to arterial puncture (*p* = 0.002), with longer times observed in the ICA-I group (4:21 h, interquartile range [IQR] 2:46 − 7:08 h) compared with the ICA-T occlusions (3:31 h, IQR 2:28 − 5:17 h). The median time from initial radiological imaging to arrival at the EVT center was also significantly longer in patients with ICA-I occlusions (2:05 h, IQR 1:22 − 3:11 h) than in those with ICA-T occlusions (1:46 h, IQR 1:15 − 2:29 h; (*p* = 0.048).

IVT was more often administered for patients with ICA-T occlusions (*p* = 0.030). There was no difference in the rate of successful recanalization (mTICI 2b-3) between the occlusion groups. Early neurological deterioration occurred numerically more often in patients with ICA-I occlusions (11%) than in those with ICA-T occlusions (8%); however, this difference did not reach statistical significance (*p* = 0.150).

**Table 1:** Demographics

	ICA-I (*n* = 356)	ICA-T (*n* = 657)	*p*-value
Median age, years (IQR)	75 (66–82)	75 (65–81)	0.500
Female	155 (43.5)	337 (51.3)	0.018
mRS 0–2 pre-stroke	287 (80.6)	529 (80.5)	0.969
mRS 3–5 pre-stroke	27 (7.6)	68 (10.4)	0.149
Hypertension	244 (68.7)	391 (60.0)	0.006
Diabetes	78 (21.9)	124 (19.0)	0.268
Median NIHSS before EVT	17 (13–20)	19 (15–21)	< 0.001
Median NIHSS after EVT	11 (4–18)	10 (4–17)	0.418
mASPECTS			0.019
10	156 (47.3)	240 (38.5)	
7–9	127 (38.5)	275 (44.1)	
5–6	29 (8.8)	82 (13.2)	
0–4	18 (5.5)	26 (4.2)	
Infarct in the posterior circulation*	31 (8.7)	35 (5.3)	0.037
Infarct in thalamus	6 (1.7)	8 (1.2)	0.543
IVT	157 (45.1)	340 (52.3)	0.030
Procedure not completed, attempt only	14 (4.0)	20 (3.1)	0.235
Median time from stroke onset to puncture, h:mm (IQR)	4:21 (2:46 − 7:08)	3:31 (2:28 − 5:17)	0.001
Median time from first imaging to EVT center arrival, h:mm (IQR)	2:05 (1:22 − 3:11)	1:46 (1:15 − 2:29)	0.048
General anesthesia	196 (55.1)	249 (37.9)	< 0.001
Conscious sedation	156 (43.8)	388 (59.1)	< 0.001
Converted from conscious sedation to general anesthesia	4 (1.1)	20 (3.0)	0.550
mTICI 2b–3	296 (83.1)	557 (84.8)	0.496
Early neurological deterioration	39 (11.0)	54 (8.2)	0.150
Post-EVT ICH	74 (20.8)	176 (26.8)	0.034
Post-EVT sICH	22 (6.2)	47 (7.2)	0.557

Data are presented as numbers (%) unless median (IQR) is indicated

Missing data were < 2% for all variables except: mRS before stroke onset (9.7%,) pre-EVT NIHSS (2.4%), post-EVT NIHSS (13.3%), and mASPECTS (4.7%)

*ICA* Internal Carotid Artery, *IQR* Inter Quartile Range, *mRS* modified Rankin Scale, *NIHSS* National Institutes of Health Stroke Scale, *mASPECTS* Modified Alberta Stroke Program Early CT Score, *IVT* Intravenous thrombolysis, *mTICI* Modified Thrombolysis in Cerebral Infarction, *sICH* Symptomatic IntraCranial Hemorrhage (ICH within 24 h resulting in NIHSS increase with minimum 4 points or death within 7 days)

*Occipital lobe, mesencephalon, pons, medulla oblongata or cerebellum

**Fig. 2:**
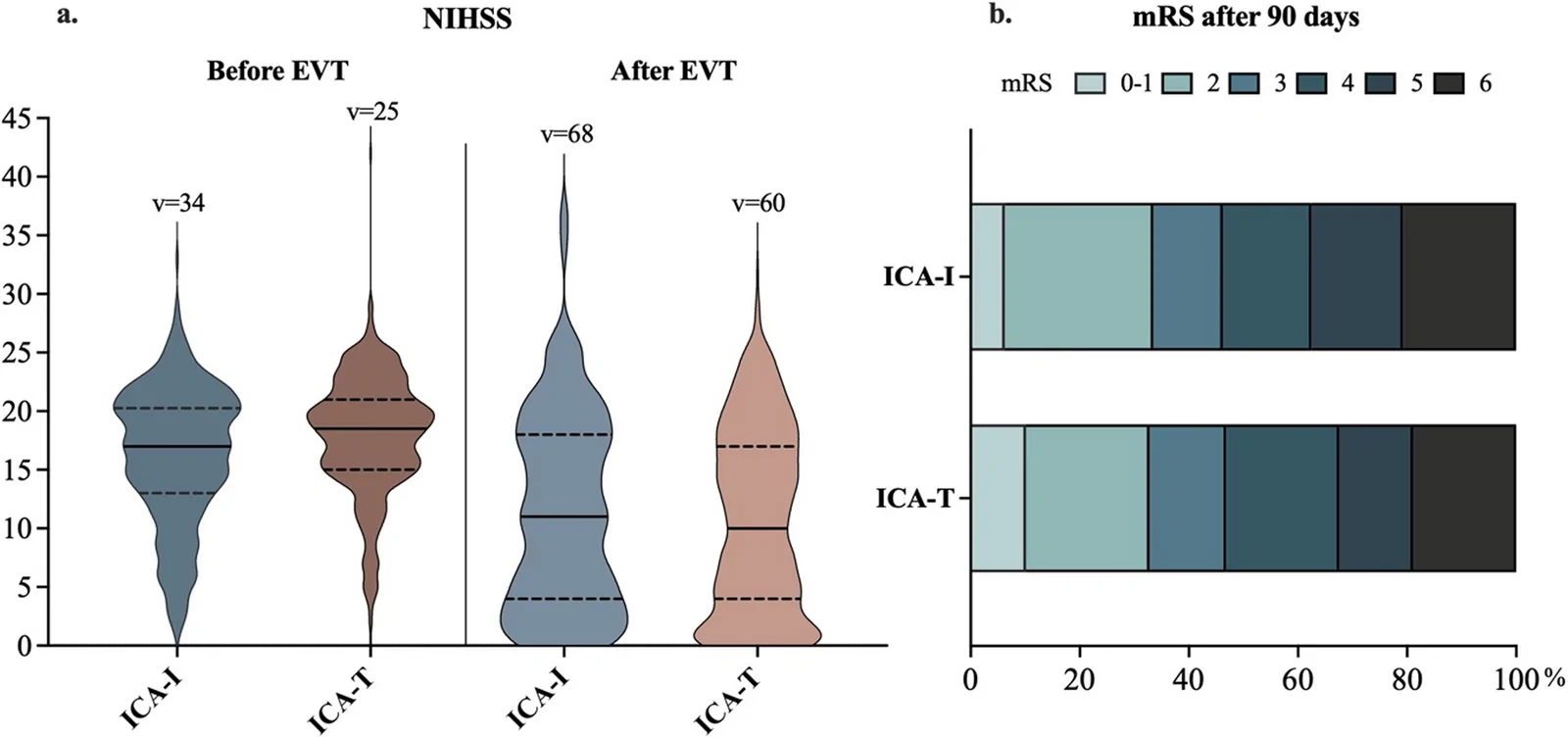
(**a**) median NIHSS with IQR and variance (v) before and after EVT and (**b**) mRS outcome after 90 days (in patients with pre-stroke mRS 0–2)

### NIHSS before EVT

The median NIHSS score before EVT was 17 in the ICA-I group (IQR: 13–20) and 19 (IQR: 15–21) in the ICA-T group (*p* < 0.001) (Fig. 2a). The variance was 34 for ICA-I occlusions and 25 for ICA-T occlusions, with a significant difference according to Levene´s test (*p* < 0.001).

### NIHSS after EVT

The median NIHSS at 24 h following EVT was 11 (IQR: 4–18) with a variance of 68 in the ICA-I group, compared to median NIHSS 10 (IQR: 4–17) with a variance of 60 in the ICA-T group (Fig. 2a). There was no significant difference in median NIHSS nor variance of NIHSS between the groups (*p* = 0.418 and *p* = 0.557).

### Functional outcome 3 months after EVT

Despite a higher proportion of patients with mRS of 0–1 at 90 days in patients with ICA-T occlusions, there were small differences in the rate of functional independence (mRS 0–2) between the occlusion groups (ICA-I 33%, ICA-T 32%, Fig. 2b), with no significant difference in the univariable logistic regression analysis (OR 0.93, 95% CI 0.69–1.24), nor in the multivariable logistic regression analysis (OR 1.00, 95% CI 0.71–1.41). Case fatality did not differ between the occlusion types (ICA-I: 21% and ICA-T: 19%).

### Subgroup analysis of anatomical variants and perfusion imaging

In the subgroup with available perfusion and angiographic imaging, 45 patients had ICA-I occlusions, of which 37 (82%) had an unbroken anterior axis and 8 (18%) a broken anterior axis. Among those with an unbroken anterior axis, 14 (38%) had a fetal type PCA while 23 (62%) did not.

**Fig. 3:**
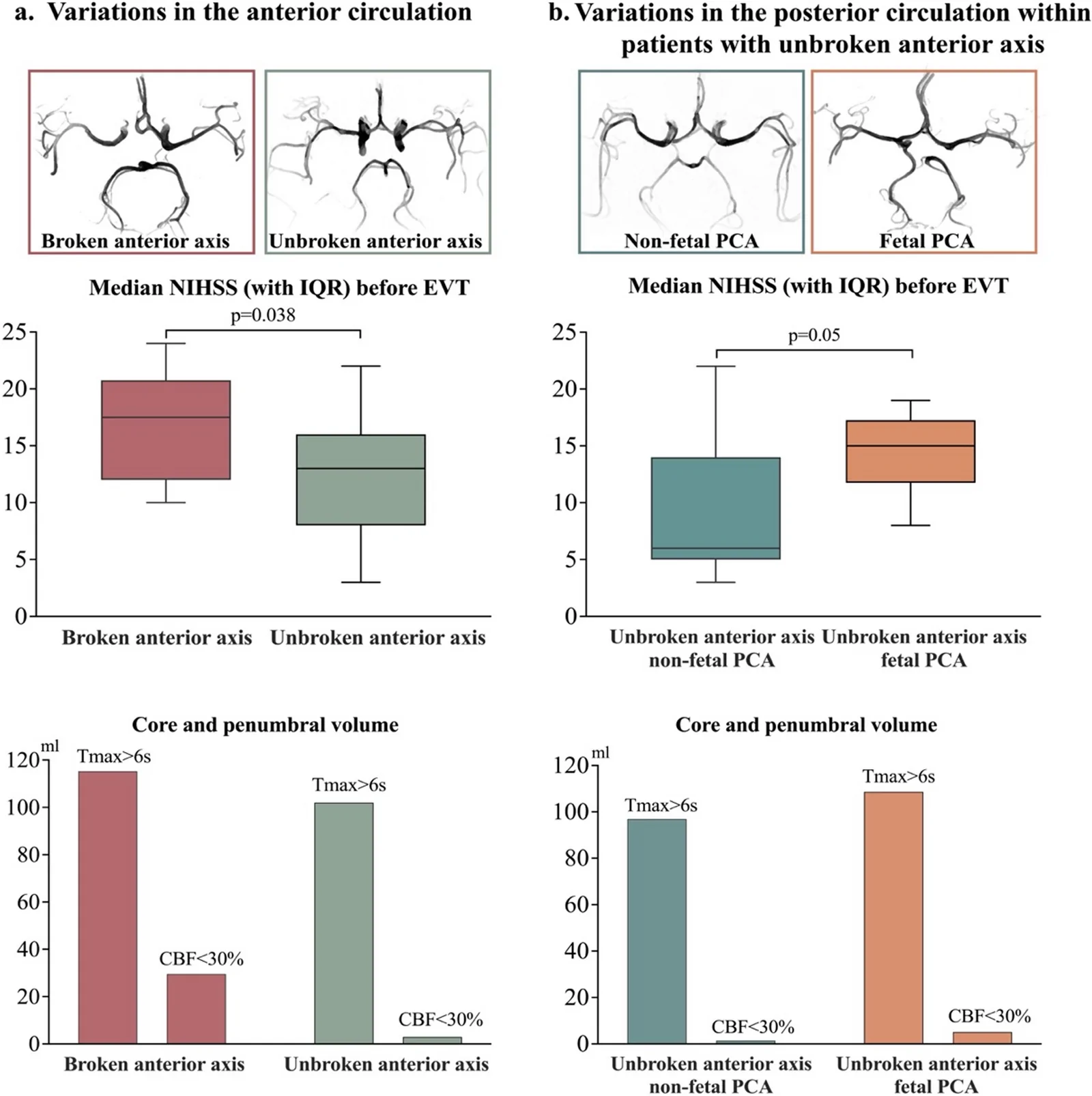
Median pre-EVT NIHSS and mean perfusion deficits for patients with ICA-I occlusions and anatomical variants in the (**a**) anterior circulation, defined as broken or unbroken axis; and (**b**) posterior circulation, defined as ipsilateral fetal type PCA or non-fetal type PCA. Perfusion volumes are presented only as means without formal testing due to small numbers in some of the subgroups with different anatomical variants

The analysis of anatomical variants in ICA-I occlusions and their association with stroke severity (Fig. 3) demonstrated a significant difference in pre-EVT NIHSS between anterior circulation variants, as assessed by the Mann-Whitney U-test. Patients with a broken anterior axis had a higher median NIHSS (17.5, IQR 12–21) compared to those with an unbroken anterior axis (median 13, IQR 8–16) (*p* = 0.038). Among patients with an unbroken anterior axis, the median NIHSS was higher for those with fetal type PCA (median 15, IQR 12–17) compared to non-fetal type PCA (median 10, IQR 6–15) but this difference did not reach statistical significance (*p* = 0.050).

**Fig. 4:**
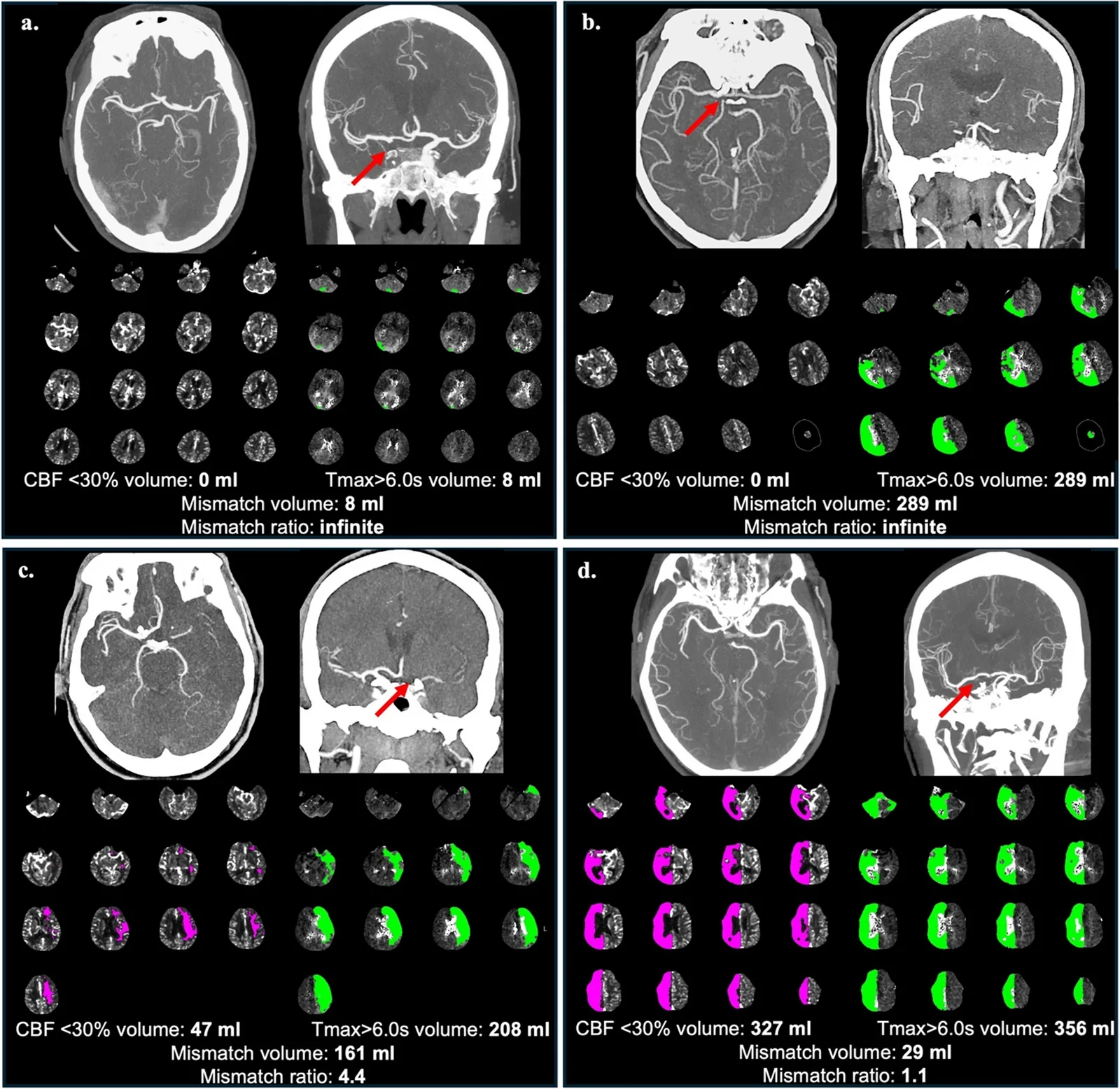
Angiographical and perfusion imaging in patients with ICA-I occlusions and different anatomical variants. Case **A**: Unbroken anterior axis and non-fetal type PCA, presenting with an NIHSS of 8, no infarct core (CBF < 30%), and a Tmax>6s volume of 8 mL. Case **B**: Ipsilateral dominant PComA/fetal type PCA, resulting in increased Tmax>6s volume involving both the MCA and the PCA vascular territories. The infarct core volume remained, however, 0 mL. This patient had an NIHSS of 13. Case **C**: Ipsilateral hypoplastic A1 segment with perfusion deficiency involving both the MCA and Anterior Cerebral Artery territories. This patient presented with an NIHSS of 19. Case **D**: Hypoplastic Anterior Communicating Artery and hypoplastic ipsilateral P1 segment, causing an extremely severe perfusion deficiency in the vascular territories of the MCA, Anterior Cerebral Artery and PCA. This patient had an NIHSS of 24, followed by rapid loss of consciousness.

Figure 4 illustrates four examples of the heterogeneity of ICA-I occlusions, with core volumes ranging from 0 mL in the MCA territory (Case A) to 327 mL with involvement of all three major vascular territories (Case D).

### Validation of initial radiology reports

This subgroup analysis included 45 patients with ICA-I occlusions and 45 patients with ICA-T occlusions. The baseline CTA radiology report was unavailable for 3 patients with ICA-I occlusions and for 2 patients with ICA-T occlusion. Of the available original reports of ICA-I occlusions 7 (17%) were written by neuroradiologists, 27 (64%) by general radiologists, 8 (19%) by radiology residents. Of the available original reports of ICA-T occlusions 14 (32%) were written by neuroradiologists, 13 (30%) by general radiologists and 16 (37%) by radiology residents.

Correct characterization of both the intra- and extracranial ICA segments was achieved in 57% of patients with ICA-T occlusions, compared with 31% of patients with ICA-I occlusions. In both groups, most reporting errors involved misclassification of both the intra- and extracranial ICA segments, and extracranial findings were frequently misinterpreted as either dissection or proximal ICA occlusion.

**Fig. 5:**
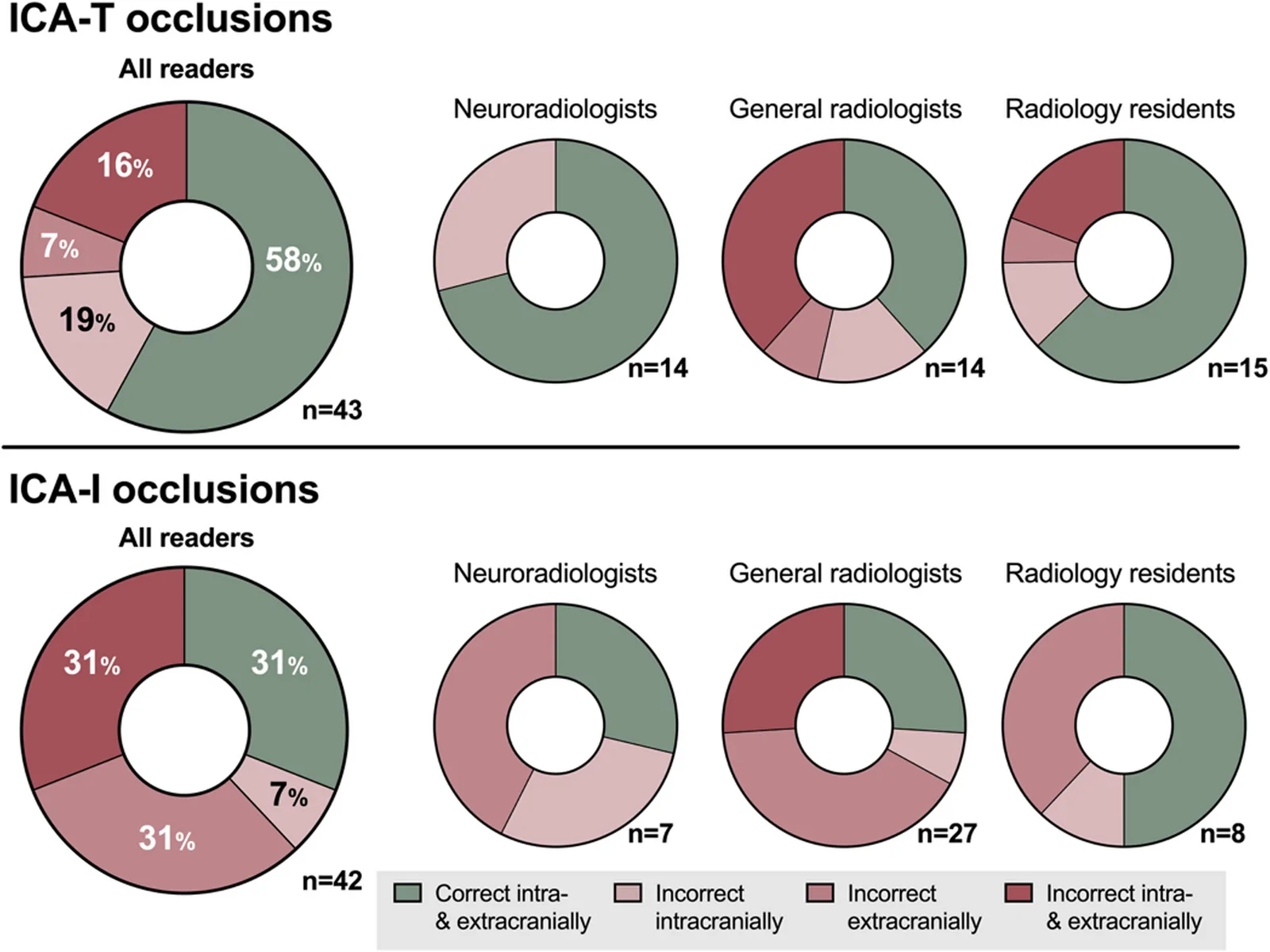
Validation of radiology reports. Doughnut charts illustrating the primary CTA interpretations in patients with ICA-I and ICA-T occlusions for all readers, and specified for readers grouped by profession as either neuroradiologists, general radiologists or radiology residents. Percentages are only given for all readers, due to the exploratory nature and small groups in the analysis stratified for profession.

In the ICA-T group, all incorrect intracranial assessments were reported as M1 occlusions (34%, *n* = 15/45). In the ICA-I group, 38% of the intracranial occlusions were not reported (*n* = 16/42, Fig. 5). In these cases, the intracranial ICA segment was either not mentioned or only described in nonspecific terms, such as “lack of contrast,” in the presence of (incorrectly) reported extracranial ICA pathology.

## Discussion

In this nationwide cohort of EVT-treated patients with intracranial ICA occlusions, ICA-I occlusions were associated with a more heterogeneous clinical and radiological presentation, longer treatment delays, and substantially higher rates of diagnostic misclassification on baseline CTA compared with ICA-T occlusions. Despite these differences, rates of successful recanalization and functional independence at 90 days were similar between the occlusion groups.

The prevalence of ICA-I occlusions among EVT-treated intracranial ICA occlusions has previously been reported to range from 16% to 30% in observational studies [5, 6, 13]. In our nationwide cohort, ICA-I occlusions accounted for 35% of all intracranial ICA occlusions. This somewhat higher proportion may partly reflect differences in case definition and the use of angiographic confirmation of the occlusion site. Furthermore, because ICA-I occlusions were frequently missed or misclassified on baseline CTA in our study, prevalence estimates based solely on initial imaging reports are likely to underestimate the true frequency of this occlusion subtype.

Previous studies have consistently shown that patients with ICA-T occlusions present with higher baseline NIHSS scores than those with ICA-I occlusions, reflecting the simultaneous compromise of both the MCA and ACA origins [5, 6]. Our findings are in agreement with these observations but further demonstrate that ICA-I occlusions exhibit substantially greater variability in NIHSS, mASPECTS, and infarct distribution. This heterogeneity is likely explained by differences in collateral circulation through the Circle of Willis [14]. Depending on the integrity of the anterior and posterior communicating pathways, ICA-I occlusions may present with either relatively mild symptoms and limited perfusion deficits (Fig. 4A) or extensive ischemia involving multiple vascular territories (Fig. 4D) or anything in between.

The higher rate of posterior circulation infarcts among patients with ICA-I occlusions may be explained by involvement of the posterior communicating artery, particularly in patients with an ipsilateral fetal-type PCA, in whom ICA-I occlusion may compromise blood flow to both the anterior and posterior circulations.

An important finding of this study was the significantly longer time from stroke onset to arterial puncture in patients with ICA-I occlusions. Although treatment delays in this subgroup have received little attention previously, our findings suggest that delayed recognition of ICA-I occlusions may contribute to these longer times. Previous studies have described the pseudo-occlusion phenomenon and the diagnostic challenges created by contrast stagnation in the cervical ICA, which may mimic extracranial ICA occlusion or dissection [7, 8]. Our results extend these observations by demonstrating that ICA-I occlusions are not only difficult to identify, but are also frequently misclassified in routine clinical practice, with only 31% correctly characterized on the initial CTA report. The most frequent reporting error was failure to recognize the intracranial occlusion, often accompanied by an erroneous diagnosis of extracranial ICA occlusion or dissection. Together, these findings suggest that increased awareness of ICA-I occlusions and their characteristic imaging appearance may improve acute stroke triage and reduce treatment delays. Perfusion imaging and artificial intelligence-based vessel occlusion detection tools may provide valuable support in cases of diagnostic uncertainty [15] and potentially help reduce delays to EVT initiation.

Despite longer treatment delays, patients with ICA-I occlusions achieved similar rates of successful recanalization and functional independence compared with ICA-T occlusions. This indicates that, within this EVT-treated cohort, the greater clinical heterogeneity and diagnostic delay associated with ICA-I occlusions did not translate into worse overall functional outcomes compared to patients with T-occlusions, but they may obviously benefit from shortening these delays.

Our findings are consistent with recent studies of isolated intracranial ICA occlusions, although direct comparisons should be interpreted cautiously because of differences in study design and patient selection [16, 17]. A subgroup analysis of the HERMES collaboration demonstrated that appropriately selected patients with isolated intracranial ICA occlusions benefit from EVT [16], whereas the ACOBOW study compared EVT with best medical treatment in patients with occlusions below the Circle of Willis and found no significant difference in functional outcome between treatment strategies [18], however the occlusions included in the ACOBOW study are located more proximal than the ICA-I occlusions in our study. In contrast, our study was not designed to compare EVT with medical management but rather to characterize the clinical and radiological heterogeneity of EVT-treated ICA-I occlusions. Consequently, our findings should not be interpreted as evidence regarding the effectiveness of EVT, but instead highlight the diagnostic challenges and collateral-dependent variability within this specific stroke subtype.

### Limitations

This study has limitations. First, because the EVAS-registry only includes patients selected for EVT, our cohort does not capture patients with ICA-I occlusions who were managed conservatively because of mild symptoms or extensive infarction. Consequently, our findings describe the spectrum of EVT-treated ICA-I occlusions rather than the entire disease population.

Secondly, although 45 ICA-I patients were included in the image analysis part, only 8 patients had a broken anterior axis, resulting in small subgroup sizes with limited statistical power and increased risk of statistical errors, and the results from these analyses should be interpreted as hypothesis generating. In addition, the perfusion imaging examples illustrate marked heterogeneity in infarct core volume, but the small number of observations limits generalizability. Similarly, the validation of initial CTA reports included a relatively small cohort and may not fully capture variability in reporting practices across radiologists and centers.

## Conclusion

Our results show that patients with ICA-I occlusions have a more heterogeneous clinical presentation, are more often overlooked in the radiological CTA assessment, and have longer treatment delays, compared with patients with ICA-T occlusions.

An increased awareness of ICA-I occlusions among radiologists may shorten door-to-puncture times and thereby improve patient outcomes.

## Data Availability

The data from that support the findings of this study are available from EVAS and Riksstroke, but restrictions apply to the availability of these data, which were used under licence for the current study and so are not publicly available. The data are, however, available upon request and with the permission of the registry holders.
